# Enrichment of SARS-CoV-2 Entry Factors and Interacting Intracellular Genes in Tissue and Circulating Immune Cells

**DOI:** 10.3390/v13091757

**Published:** 2021-09-02

**Authors:** Abhinandan Devaprasad, Aridaman Pandit

**Affiliations:** 1 Center for Translational Immunology, University Medical Center Utrecht, 3584 EA Utrecht, The Netherlands; abhi2308@gmail.com; 2 Department of Rheumatology & Clinical Immunology, University Medical Center Utrecht, 3584 CX Utrecht, The Netherlands

**Keywords:** SARS-CoV-2, ACE2, immune cells, gene enrichment, T cells, macrophages, dendritic cells

## Abstract

SARS-CoV-2 uses ACE2 and TMPRSS2 to gain entry into the cell. However, recent studies have shown that SARS-CoV-2 may use additional host factors that are required for the viral lifecycle. Here we used publicly available datasets, CoV-associated genes, and machine learning algorithms to explore the SARS-CoV-2 interaction landscape in different tissues. We found that in general a small fraction of cells express ACE2 in the different tissues, including nasal, bronchi, and lungs. We show that a small fraction of immune cells (including T cells, macrophages, dendritic cells) found in tissues also express ACE2. We show that healthy circulating immune cells do not express ACE2 and TMPRSS2. However, a small fraction of circulating immune cells (including dendritic cells, monocytes, T cells) in the PBMC of COVID-19 patients express ACE2 and TMPRSS2. Additionally, we found that a large spectrum of cells (in tissues and circulation) in both healthy and COVID-19-positive patients were significantly enriched for SARS-CoV-2 factors, such as those associated with RHOA and RAB GTPases, mRNA translation proteins, COPI- and COPII-mediated transport, and integrins. Thus, we propose that further research is needed to explore if SARS-CoV-2 can directly infect tissue and circulating immune cells to better understand the virus’ mechanism of action.

## 1. Introduction

Severe acute respiratory syndrome coronavirus 2 (SARS-CoV-2) is a novel virus from the Coronaviridae family that has infected more than 100 million individuals and has caused a rapidly unfolding global pandemic. A large number of infected individuals present no or mild symptoms and yet can spread the virus to others. However, some infected individuals develop severe acute respiratory distress syndrome resulting in coronavirus disease (COVID-19). This leads to a unique dysregulation of the immune system that is accompanied by a strong inflammatory response, cytokine storm, and ultimately respiratory distress and viral sepsis [1]. However, the reason why the immune system enters such dysregulation is not yet clear. As a result, the exponential growth of the infections and failure to restrain the infections has brought the world to a standstill. Thus, there is an urgent need to understand the mechanisms of infection and pathophysiology of SARS-CoV-2.

SARS-CoV-2 belongs to Betacoronavirus genera of Coronaviridae family, and two other Betacoronaviruses (SARS-CoV and MERS-CoV) are known to infect humans. SARS-CoV-2 differs from the other two Betacoronaviruses and results in milder clinical manifestation in most individuals but has a high transmission rate between humans [2]. SARS-CoV-2 uses spike protein S to infect human cells. The S protein of SARS-CoV-2 is homologous to the spike protein of SARS-CoV (>75% sequence identity) [3]. The S protein of SARS-CoV has been shown to bind to a human metallopeptidase receptor called angiotensin-converting enzyme 2 (ACE2) for cell entry [4] and requires a target cell serine protease called transmembrane protease serine 2 (TMPRSS2) for its proteolytic priming [5,6,7]. Due to the high sequence similarity in spike proteins of the two SARS coronaviruses, SARS-CoV-2 was postulated to use ACE2 as the entry receptor [3]. Multiple studies have now confirmed that the S protein of SARS-CoV-2 binds to human ACE2 for cellular entry and TMPRSS2 aids in its proteolytic activation [8,9,10]. Contrary to SARS-CoV, FURIN (PCSK3), a proprotein convertase was found to activate the S protein of SARS-CoV-2 by cleaving at the FURIN cleavage site found between the S1/S2 domains of the S protein [11]. FURIN-dependent activation along with TMPRSS2 and lysosomal cathepsins had cumulative effects on viral entry [11]. Furthermore, genome-wide association studies have shown that FURIN and TMPRSS2 had significant genetic variants prevalent in COVID-19 patients, suspecting a role in susceptibility to infection [12]. However, none were found in ACE2, and the exact function of these variants in the binding of SARS-CoV-2 remains to be explored [13].

The presence of these entry factors, specifically ACE2 and TMPRSS2, has been detected in bronchial and nasal epithelium and alveolar epithelial type II cells of the respiratory tract and in cells from the ileum and colon of the digestive tract [14,15,16,17,18,19]. Hou et al. reported a decreasing gradient in gene expression of ACE2 and infectivity of SARS-CoV-2 from the proximal to distal respiratory tract, with the ciliated airway and AT2 cells being the main target for infection. Pathological changes due to SARS-CoV-2 have been reported to be found in lung, kidney, heart, blood vessels, liver, colon, and digestive tract [1,20,21]. Using single-cell RNA-Seq datasets of 13 human tissues, Qi et al. reported expression of ACE2 in lung, liver, stomach, ileum, kidney, and colon. Qi et al. postulated ANPEP, DPP4, and ENPEP as candidate co-receptors, as they were co-expressed with ACE2 in tissue [18]. The co-expression of ACE2 and DPP4 was experimentally validated independently by Amati et al. in the nasopharyngeal and oropharyngeal swabs of COVID-19-positive patients [22]. Sungnak et al. analyzed multiple single-cell RNA-Seq datasets of 17 different tissues and found ACE2 expression limited to airways, cornea, esophagus, ileum, colon, liver, gallbladder, heart, kidney, and testis [23]. While TMPRSS2 was expressed by a broader number of cell types and tissues, the cells co-expressing ACE2 and TMPRSS2 were found to be from the respiratory tree, cornea, esophagus, ileum, colon, gallbladder, and common bile duct. However, several innate immune genes, such as IDO1, IRAK3, NOS2, TNFSF10, OAS1, and MX1, were found to be co-expressed with ACE2 in respiratory epithelial cells. In lung and bronchial tissue datasets, FURIN was found to be expressed in bronchial transient secretory cells and also in both ACE2+/TMPRSS2+ and ACE2+/TMPRSS− cells, thus reducing the proteolytic dependence of TMPRSS2 [24].

However, most studies have focused on ACE2, TMPRSS2, and FURIN. We still do not fully understand all the mechanisms by which SARS-CoV-2 infects and interacts with human cells. Recently, using cell lines and mice experiments, the interaction between the host CD147 and SARS-CoV-2 spike protein was found to be an alternative route of viral entry into host cells via endocytosis [25]. However, this was later shown to be incorrect, with no evidence of CD147 being involved as the binding receptor for SARS-CoV-2 spike protein [26]. Two seminal studies, Zhou et al. and Gordon et al., have looked into genes associated with SARS-CoV-2 to gain further insights into the mechanisms of action of the virus and to find potential (intra-)cellular targets for future therapies [27,28]. Zhou et al. took a network medicine approach to repurpose drugs for SARS-CoV-2 and by studying the host factors that have been associated with previously known coronaviruses (four human coronaviruses: SARS-CoV, MERS-CoV, HCoV-229E, and HCoV-NL63; one murine coronavirus; and one avian coronavirus) [28]. Gordon et al. cloned and expressed 29 SARS-CoV-2 proteins in human cells and applied affinity purification mass spectrometry to identify 332 high-confidence human proteins that interact with SARS-CoV-2 proteins [27]. Studying human SARS-CoV-2-specific protein–protein interactions aims to provide insights into host factors that may influence infection dynamics and provide potential therapeutic targets.

Despite the growing literature on SARS-CoV-2 infection, it is still not clear which cell types are infected by the virus. Most studies have considered the expression of ACE2 within a cell type as the determining factor of SARS-CoV-2 susceptibility. However, it is still not clear if the human cells that express ACE2 also express the virus-associated genes in them. Additionally, it is now clear that immune cells play a crucial role in the infection dynamics of SARS-CoV-2 and that the underlying dysregulation of the immune system is distinct from other coronaviruses [29]. Boumaza et al. found that SARS-CoV-2 infects circulating monocytes and macrophages that further drive immunoparalysis and advance disease progression [30]. Additionally, two studies in preprint have shown that immune cells can be infected by SARS-CoV-2. One such study showed that immune cells (CD4+ T cells, CD8+ T cells, B cells, and monocytes) in peripheral blood mononuclear cells (PBMCs) and lung tissue were infected with SARS-CoV-2 and that infection was independent of ACE2 expression [31]. In another study, ACE2-expressing tissue resident CD169+ macrophages were found to be infected by SARS-CoV-2 in the spleen and lymph nodes, which further progressed neutralization of the tissue [32]. Such studies are essential to shed light on the role of immune cells in the disease’s progression. With the limited number of such studies focusing on fewer immune cells, it is still not clear which all-tissue and circulating immune cells are susceptible to infection and can potentially support the viral infection. To address this problem, we built upon the results obtained from the study of Sungnak et al. not only to explore ACE2 and TMPRSS2 expression but also to explore the expression of SARS-CoV-2-associated host factors in several tissues and cell types, including lung, kidney, and immune cells, using publicly available single-cell RNA-Seq data. We additionally extended this approach to study circulating immune cells curated from bulk RNA-Seq datasets. We also included a single-cell RNA-Seq dataset of PBMCs from patients with varying severity of COVID-19 to explore the expression of SARS-CoV-2-associated host factors in infected cells.

## 2. Materials and Methods

### 2.1. SARS-CoV-2-Associated Gene Sets

In this study, we used five different publicly available gene lists. The Zhou gene list taken from the Zhou et al. study consists of 119 genes involved in the virus–host protein interactions of 4 HCoVs (HCoV-229E, HCoV-NL63, MERS-CoV, and SARS-CoV), a murine beta coronavirus (MHV), and an avian infectious bronchitis coronavirus (IBV) [28]. The phylogenetic analysis of the CoV genomes reveals that SARS-CoV is 79.7% similar in nucleotide sequence to that of SARS-CoV-2 and that the MHV cluster is in the same lineage as HCoV-HKU1 and HCoV-OC43, while the IBV cluster is in the same lineage as HCoV-229E and HCoV-NL63. Hence, inclusion of SARS-CoV, MHV, and IBV is crucial to exploring the virus–host protein interactions of SARS-CoV-2. While the Zhou gene list is the virus–host protein interactions of the older HCoV (SARS-CoV and SARS-CoV-like coronaviruses), the Gordon gene list taken from the Gordon et al. study captures the virus–host protein interactions of the novel SARS-CoV-2. The Gordon gene list consists of 332 genes encoding the host proteins of an HEK-293T cell line that are physically associated with 26 of the SARS-CoV-2 proteins identified using affinity-purification mass spectrometry [27]. The 28-EF gene list curated by Singh et al. from literature mining consists of 28 human genes that facilitate and restrict viral entry of SARS-CoV-2, SARS-CoV, MERS-CoV, hCoV-229E, and hCoV-NL63 [33]. The integrin gene list comprises all known human integrins [34]. We have included the integrin gene list based on the hypothesis that the RGD (arginine-glycine-aspartate) motif present in the viral spike protein is commonly used by viruses to bind to integrins on the host cell surface and that this may be an alternative route of viral entry [35]. Lastly, we extracted the recently available SARS-CoV-2 interactome from the Stukalov et al. study that infected A549 cells with SARS-CoV-2 proteins and profiled the virus–host interacting proteins using affinity purification and mass spectrometry [36]. The Stukalov gene list is composed of 876 proteins that are specific to SARS-CoV-2 interactome.

### 2.2. Single-Cell Data Analysis

**Figure 1:**
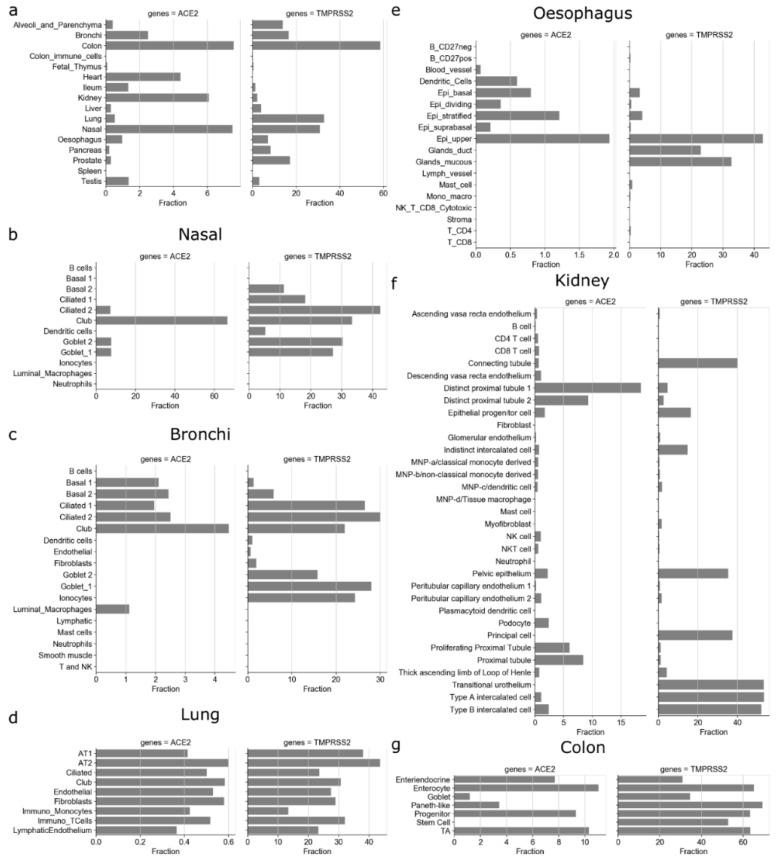
Fraction of ACE2- and TMPRSS2-expressing cells. (**a**) Fraction of cells (as percentage on *y* axis) that express ACE2 and TMPRSS2 in different tissues. Fraction of cells within tissue type: (**b**) nasal, (**c**) bronchi, (**d**) lung, (**e**) esophagus, (**f**) kidney, and (**g**) colon that expresses ACE2 and TMPRSS2.

**Figure 2:**
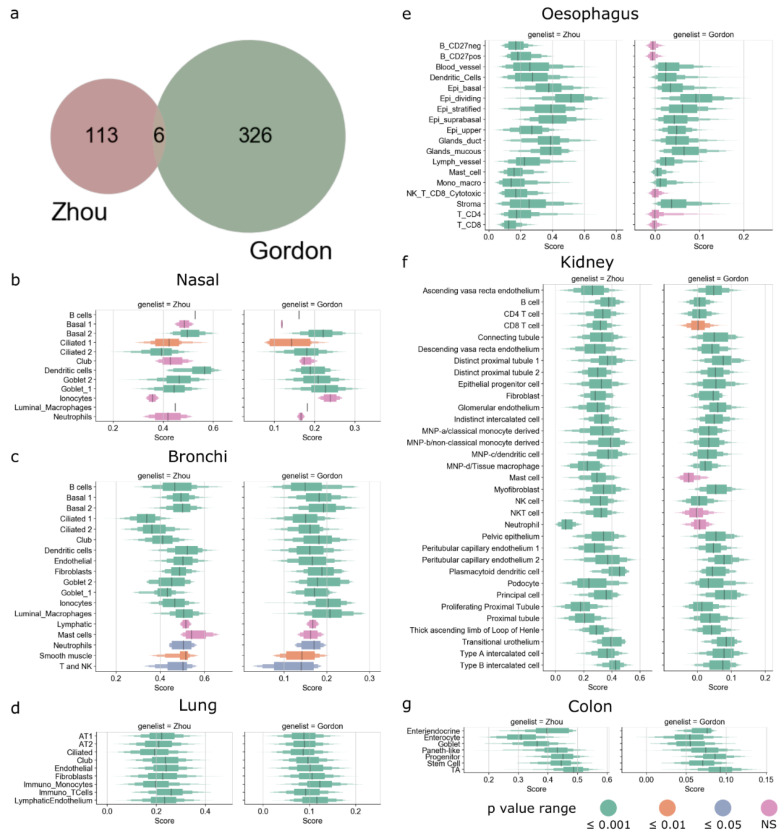
SARS-CoV-2 host factor genes. (**a**) Venn diagram showing overlap of SARS-CoV-2 host factor genes between the Zhou and Gordon gene lists. Boxplot showing the distribution of gene score of Zhou and Gordon genes for different cell types: (**b**) nasal, (**c**) bronchi, (**d**) lung, (**e**) esophagus, (**f**) kidney, and (**g**) colon. Black line represents median, height of box corresponds to number of cells in score range. Color of the box corresponds to the Wilcoxon *p* value computed with the alternative set to >0. See bottom right of Figure 2 for *p* value range.

The normalized and preprocessed single-cell RNA-Seq datasets were acquired from the COVID-19 cell atlas (www.covid19cellatlas.org, accessed date: 30 August 2021). The normalization, preprocess steps, and cell type annotation of all the single-cell RNA-Seq datasets performed using the Scanpy suite have been described previously [23]. The total fraction (Figure 1a) was calculated in percentage for all cells in a dataset for all datasets. The fraction (Figure 1b–g) was calculated in percentage for each annotated cell type for all datasets. The gene score (Figure 2b–g) for each of the above gene lists and for all cell types in all datasets was calculated using the ‘tl.score_gene’ function from the Scanpy (Single-cell analysis in python) suite [37]. The gene score is the difference in the average expression of the gene list to the average expression of background (all other) genes. This score reproduces the gene score in Seurat pipeline [38]. Positive gene scores show that the cell expresses the given set of genes higher than the background. Wilcoxon non-parametric statistical analysis was performed with the alternative set to ‘>0’ on the gene scores for every cell type in all datasets to measure the significance of gene score. The *p* values were binned in ranges of values ≤0.001, ≤0.01, ≤0.05, and NS (non-significant, when *p* value > 0.05). As a control, we computed gene scores and performed Wilcoxon non-parametric statistical tests using randomly selected genes. We did not find significant enrichment of randomly selected genes in the majority of tissues and cell types (data not shown).

### 2.3. DIME on Immunome (Bulk RNA-Seq)

**Figure 3:**
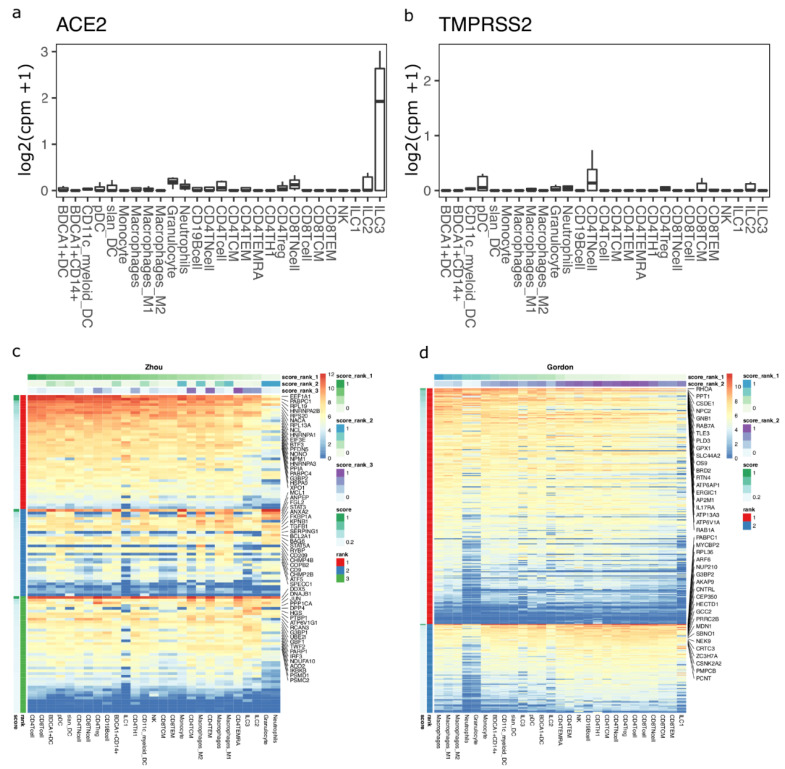
DIME enrichment of SARS-CoV-2 host factors in circulating immune cells. Expression of (**a**) ACE2 and (**b**) TMPRSS2 in circulating immune cells from the bulk dataset. Expression values are in log2(cpm + 1). DIME heatmap showing ranked enrichment of (**c**) Zhou, and (**d**) Gordon gene list in the circulating immune cells. The ranks depict the clusters as identified by DIME (see methods). Top 20 genes for each rank are shown. The cells are ordered based on the score of the rank 1 (top-weighted rank). Expression values are in log2(cpm + 1).

The DIME tool [39] identifies the top gene (from an input gene list) and top cell type cluster within an expression dataset by using non-negative matrix factorization (NMF). The shiny app implementation of the DIME tool is available on bitbucket for installation and use (https://bitbucket.org/systemsimmunology/dime/src/master/; accessed date: 30 August 2021). The DIME was applied on the immunome dataset available as a default expression dataset in the tool. The immunome dataset comprises bulk RNA-Seq gene expression data of 27 immune cells, of which 11 are myeloid, and 16 are lymphoid. All datasets used in the construction of the immunome are from publicly available datasets [39]. The cells used here are from unstimulated (except for macrophages, which were monocyte-derived) healthy donors. The DIME was run on the immunome using the Zhou, Gordon, 28-EF, and integrin gene lists to identify key cell types important for these gene lists (Figure 3). The highest ranking cluster was identified using Frobenius norm [39]. The top 25 genes for each ranking cluster are displayed (Figure 3). Reactome pathway enrichment analysis was performed on genes in the top 25th percentile in each ranking cluster for the DIME results of the different gene lists (Figure S5).

### 2.4. Protein Expression of the SARS-CoV-2-Associated Genes in Circulating Immune Cells and Tissues

To check the protein expression of the key SARS-CoV-2-associated genes identified by DIME (Figure 3 and Figure S4), we used two publicly available protein expression datasets—namely, immprot and the human protein atlas (HPA). The immprot dataset comprises protein expression of 28 circulating primary human hematopoietic cell populations from healthy donors identified by high-resolution mass spectrometry-based proteomics [40]. The HPA dataset comprises protein expression of cells (mostly non-immune cell types) from 32 tissues [41]. We checked the protein expression of genes in the top 25th percentile of each cluster from the DIME results of the SARS-CoV-2-associated gene lists in the two protein expression datasets. Additionally, we also computed the Pearson correlation between the RNA-Seq and protein datasets for the cells that were common between the two datasets.

### 2.5. Plotting and Visualization

DIME was implemented and visualized in R (version = 3.6.1). The single-cell RNA-Seq analysis was performed in python (version = 3.6) using the Scanpy package and Wilcoxon statistics using the stat package from python.

## 3. Results

### 3.1. ACE2-Expressing Cells across Different Single-Cell RNA-Seq

We first assessed the expression of ACE2 and TMPRSS2 in different tissues and cell types using 16 single-cell RNA-Seq datasets. The ACE2 gene is expressed only in a small fraction of cells in different tissues, among which colon (7.55%), nasal (7.47%), kidney (6%), heart (4.4%), bronchi (2.49%), testis (1.35%), and ileum (1.33%) have the highest fraction of ACE2-expressing cells (Figure 1a). Despite being one of the most affected tissues, only 0.52% of cells expressed ACE2 in the lung. In contrast, TMPRSS2 is expressed in many more cells in different tissues: colon (58.58%), lung (32.83%), nasal (30.87%), prostate (17.1%), bronchi (16.53%), alveoli and parenchyma (13.8%). Interestingly, the ACE2 gene was expressed in less than 0.01% of spleen and colon immune cells (Figure 1a).

We further studied the expression of ACE2 and TMPRSS2 in different cell types in each of the tissues (Figure 1b–g and Figure S1). The decreasing gradient of ACE2 expression was observed in the respiratory tract from nasal to bronchi to lung to alveoli and parenchyma (Figure 1a–d). These results are in corroboration with the findings from Hou et al. [15]. The club cells were the highest fraction of ACE2-expressing cells in nasal (66.6%) and bronchi (4.46%). Interestingly, the fraction of TMPRSS2-expressing ciliated-1 cells showed an increasing trend from nasal (18.18%) to bronchi (26.47%). We found that in lungs, only a small fraction (0.36%–0.6%) of cells for each cell type expressed the ACE2 gene (Figure 1d). For example, 0.41% of AT1 (alveolar type I) cells and 0.6% of AT2 (alveolar type II) cells expressed the ACE2 gene (Figure 1d), while more than one-third of AT1 (38%) and AT2 cells (43.68%) expressed TMPRSS2. The low ACE2 expression in lungs has also been reported in other studies [17,18].

Other organs that are known to undergo pathological changes due to SARS-CoV-2 include the kidney, heart, vessels, liver, colon, and digestive tract [1,20,21,42]. In the esophagus, a part of the upper gastrointestinal (GI) tract of the digestive system, the fraction of cells expressing ACE2 included 1.93% of the upper and 1.21% of stratified epithelial cells (Figure 1e). Colon had the highest fraction of ACE2-expressing cells, from the progenitors (9.3%) and transit-amplifying (TA) cells (10.31%) to the differentiated enterocytes (11%) expressing ACE2 (Figure 1g). Overall, the ACE2-expressing cells (Figure 1a) were lower in the esophagus (0.97%) compared with tissues of the lower GI tract, such as ileum (1.33%) and colon (7.55%). ACE2 was expressed at higher frequency in renal tubular cells, such as distinct proximal tubule 1 (18.46% cells), distinct proximal tubule 2 (9.27% cells), and proximal tubule (8.45% cells), and SARS-CoV-2 has been shown to damage kidney in patients (Figure 1f) [20]. Similarly, a small fraction of cells in each cell type found in heart (<18% stromal cells, 3.26% adipocytes, etc.), colon (7.7% of enteroendocrine, 1.17% goblet cells, etc.), and ileum (0.24% endothelium, 0.86% goblets, etc.) expressed the ACE2 gene (Figure S1). Interestingly, for the same/similar cell types found in different tissues and organs, we found different fractions of them to be positive for ACE2 expression. For example, 13% of fibroblasts expressed the ACE2 gene in heart, while only 0.2% and 0.17% of fibroblasts expressed the ACE2 gene in alveoli and ileum, respectively (Figure S1). Similarly, 41.65% of enterocytes expressed the ACE2 gene in ileum, while 11% of enterocytes expressed the ACE2 gene in colon (Figure 1 and Figure S1). This potentially indicates that cells with the same/similar phenotype might vary in terms of ACE2 expression based on the local tissue environment.

### 3.2. Enrichment of SARS-CoV-2-Associated Genes in Tissues and Cells

We next studied the expression of SARS-CoV-2-associated genes and their expression profiles in different tissues and cell types. Only six genes (G3BP1, G3BP2, MARK3, PABPC1, PABPC4, and SRP54) overlapped between SARS-CoV-2-associated genes proposed by Gordon et al. and multiple CoVs-associated genes proposed by Zhou et al. (Figure 2a and Figure S2) [27,28]. Enrichment analysis revealed that the overlapping genes belonged to the ribonucleoprotein complex (GO:1990904) and stress granule assembly (GO:0010494) GO terms. However, due to the stark differences between the gene lists of SARS-CoV-2 and other coronavirus-associated genes, we considered both these gene lists for further analysis.

Next, we calculated the gene score for both Gordon and Zhou gene lists in each cell type in each tissue from single-cell RNA-Seq datasets. A positive gene score indicates that the genes in the list show higher gene expression than the background by the cell type. Interestingly, tissue-resident immune cells, such as dendritic cells and luminal macrophages in nasal and bronchi (Figure 2b,c), monocytes and T cells in lung (Figure 2d), dendritic cells in esophagus (Figure 2e), several lymphoid and myeloid cells in kidney (Figure 2f), exhibited significantly positive gene scores for SARS-CoV-2 and CoV-associated genes. This potentially indicates that the host factors associated with coronaviruses are ubiquitously expressed by human immune cells (see Figure 2 and Figure S3 for all single-cell RNA-Seq datasets).

### 3.3. Circulating Immune Cells as Potential Targets of SARS-CoV-2

To test if immune cells can potentially act as targets of SARS-CoV-2, we studied ACE2 and TMPRSS2 expression in circulating immune cells using bulk transcriptomics data. We found that ACE2 and TMPRSS2 are not expressed in any circulating immune cells (Figure 3a,b). We next performed machine learning-based gene enrichment analysis for CoV- and SARS-CoV-2-associated genes in bulk immune cell transcriptomics datasets using DIME (see methods). DIME is a tool specifically built for identifying key immune cell types and their corresponding key genes from a user-defined gene list [43]. We found that several CoV-associated genes from the Zhou gene list were highly expressed in T cells, and several of these genes were expressed in nearly all immune cell types (Figure 3c; see colors for score rank 1). The genes that were highly expressed in all immune cells included EEF1A1, PABPC1, RPL19, HNRNPA2B1, etc., and these genes correspond to pathways related to mRNA translation (Figure S5a). The NSP-1 protein of CoV [44] and SARS-CoV-2 [45] has been shown to be involved in immune evasion by shutting down RNA translation. The exact interaction of SARS-CoV-2 and the genes involved in RNA translation in T cells and other immune cells is unknown and needs to be further elucidated to see if there is a link between this interaction and the lymphopenia often seen in COVID-19 patients [46]. Similarly, we found that macrophages and T cells were enriched in SARS-CoV-2-associated genes from the Gordon gene list (Figure 3d; see colors for score rank 1 and 2). The key genes enriched were found to be those associated with RAB signaling and neutrophil degranulation (Figure S5b). RABS are small GTPases that regulate vesicular transport, and viruses such as HIV have been shown to use RAB signaling-related host vesicular transport for viral assembly [47]. The exact involvement of RAB signaling in SARS-CoV-2 viral assembly is yet to be explored.

### 3.4. Immune Cells in Tissue as Potential Targets of SARS-CoV-2

On the contrary, in multiple different single-cell RNA-Seq datasets from tissues, a fraction of different immune cell types expressed ACE2 and TMPRSS2 genes (Figure 1b–d and Figure S1). Specifically, 0.42% of monocytes in lungs expressed the ACE2 gene, and this fraction is comparable with the other lung cells, such as AT2, AT1, and endothelial cells (Figure 1d). Similarly, 0.45% to 0.54% of monocyte-derived phagocytes (dendritic cells and macrophages) in kidney, 0.12% of macrophages in ileum, and 0.59% of dendritic cells in esophagus expressed the ACE2 gene (Figure 1e,f). Interestingly, lymphocytes in alveoli, pancreas, esophagus, and spleen did not express the ACE2 gene, but a small fraction of T cells in kidney (0.7% of CD8 + T cells, 0.49% of CD4 + T cells, 0.98% of NK cells, and 0.1% of B cells), colon (0.03% CD8 + T cells and 0.02% of memory B cells), lung (0.51% of T cells), ileum (0.12% of T cells), and heart (0.16% of T/NK cells and 0.09% B cells) expressed the ACE2 gene (Figure S1). Although the fraction of ACE2-expressing immune cells is very small, it is comparable with the fractions observed for some of the cell types known to be infected by SARS-CoV-2, such as AT2, AT1, epithelial, and endothelial cells. In summary, we found that immune cells in tissues (Figure 2) and circulation (Figure 3) are enriched for CoV- and SARS-CoV-2-associated genes.

### 3.5. Protein Expression of the Key SARS-CoV-2-Associated Genes Found in Circulating Immune Cells and in Tissues

We found protein expression of all the key (top 25 percent of genes of all clusters from DIME) SARS-CoV-2-associated genes to be high in all the circulating immune cells, Figure S7a. The Pearson correlation estimate between the RNA-Seq and protein data (of circulating immune cells) for five cell types was found to be >0.4 for all cell types except CD4 naive T cell (*r* = 0.32). The correlations were found to be statistically significant (*p* value < 0.05) for all the cell types (Figure S7b). In the tissue cells, we observed that all the key genes except the integrins were expressed ubiquitously across all the tissues except the brain, endometrium, kidney, liver, skeletal muscle, spleen, and thyroid glands (Figure S8).

### 3.6. Enrichment of SARS-CoV-2-Associated Genes in the PBMCs of COVID-19 Patients

**Figure 4:**
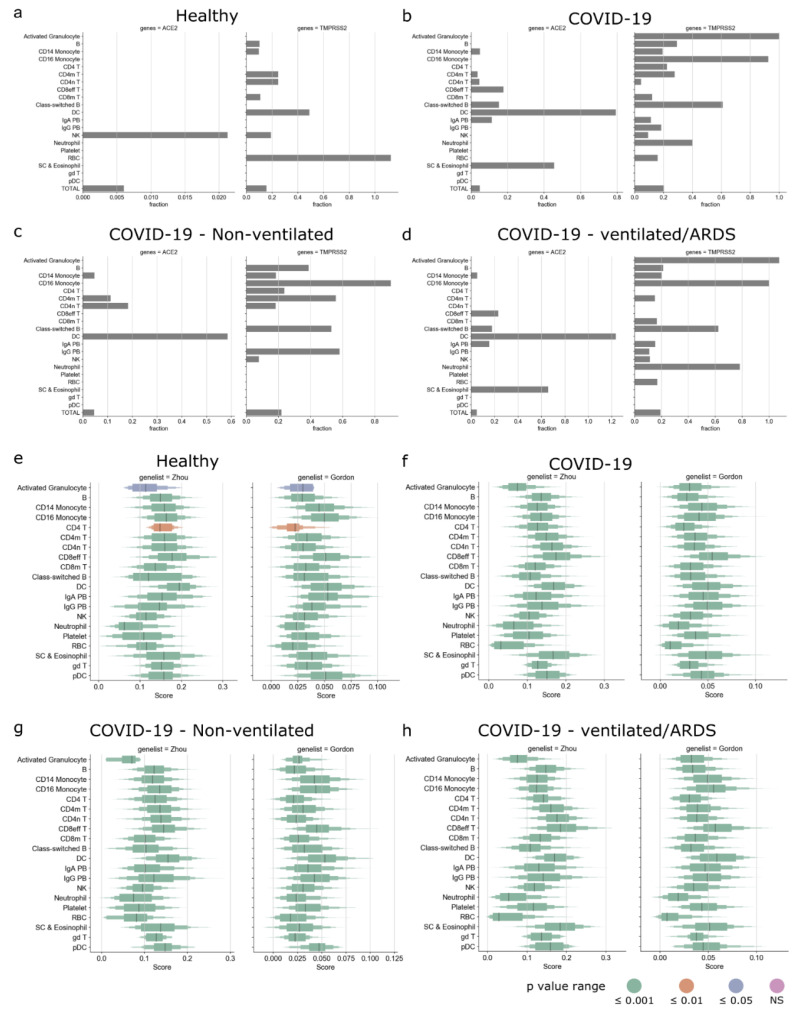
Enrichment of SARS-CoV-2 host factors in PBMCs of COVID-19 patients. Expression of ACE2 and TMPRSS2 in the pooled (**a**) healthy controls, (**b**) all COVID-19 patients, (**c**) non-ventilated COVID-19 patients, and (**d**) COVID-19 patients that developed ARDS. Boxplot showing distribution of gene score of Zhou and Gordon genes for (**e**) healthy controls, (**f**) all COVID-19 patients, (**g**) non-ventilated COVID-19 patients, and (**h**) COVID-19 patients that developed ARDS and were on ventilator.

We first explored the expression of SARS-CoV-2-associated genes in the PBMCs of the healthy controls and COVID-19 patients. Here, we used the published single-cell RNA-Seq dataset from the COVID-19 cell atlas [48,49]. The Wilk et al., data comprised single-cell RNA-Seq of PBMCs taken from seven COVID-19 patients (confirmed by positive SARS-CoV-2 nasopharyngeal swab by RT-PCR) and six healthy controls. We then studied the expression of ACE2 and TMPRSS2 across pooled healthy controls and the different types of COVID-19 patients (Figure 4a–d). In the pooled healthy controls, only a small fraction of NK cells (0.021%) expressed ACE2, whereas TMPRSS2 was expressed by several immune cells, such as B cells, CD14 monocytes, CD4 memory and naive T cells, dendritic cells, and NK cells (Figure 4a). In the pooled COVID-19 patients, ACE2 was expressed in several immune cells, such as dendritic cells, CD14 monocytes, CD4 memory T cells, CD4 naive T cells, CD8 effector T cells, etc.; TMPRSS2 was expressed in most immune cells (Figure 4b). Interestingly, in the pooled COVID-19 patients, dendritic cells expressed ACE2 but not TMPRSS2. In the COVID-19 patients who did not require a ventilator, ACE2 was expressed in CD14 monocytes, CD4 memory and naive T cells, and dendritic cells; TMPRSS2 was expressed in several immune cells (Figure 4c). However, in the COVID-19 patients that developed acute respiratory distress syndrome (ARDS) and required a ventilator, ACE2 was expressed in CD14 monocytes, CD8 effector T cells, dendritic cells, stem cells, and eosinophils (Figure 4d).

We then calculated the gene score for both Gordon and Zhou gene lists for the pooled healthy controls and the different types of COVID-19 patients. The gene scores of Gordon and Zhou gene lists were significantly positive for all cells in the pooled healthy controls and the different types of COVID-19 patients (Figure 4e–h). Myeloid cells such as dendritic cells, CD14 and CD16 monocytes, plasmacytoid dendritic cells, and lymphoid cells such as CD8 effector T cells and gamma delta T cells had high gene scores across all types of patients. We then explored if the above patterns were discernable in the individual patient samples (Figure S9a). We found that the fraction of ACE2- and TMPRSS2-expressing cells was heterogenous and did not show any patterns corresponding to the clinical outcome (Figure S9b–i). However, upon inspection of the gene scores, we observed that the gene scores for dendritic cells were consistently high across all patients (Figure S9j–q), indicating that dendritic cells may play a key role in the infection dynamics.

### 3.7. Enrichment of Human Lung Cell Line-Derived Virus–Host Interactome in Circulating and Tissue Immune Cells

**Figure 5:**
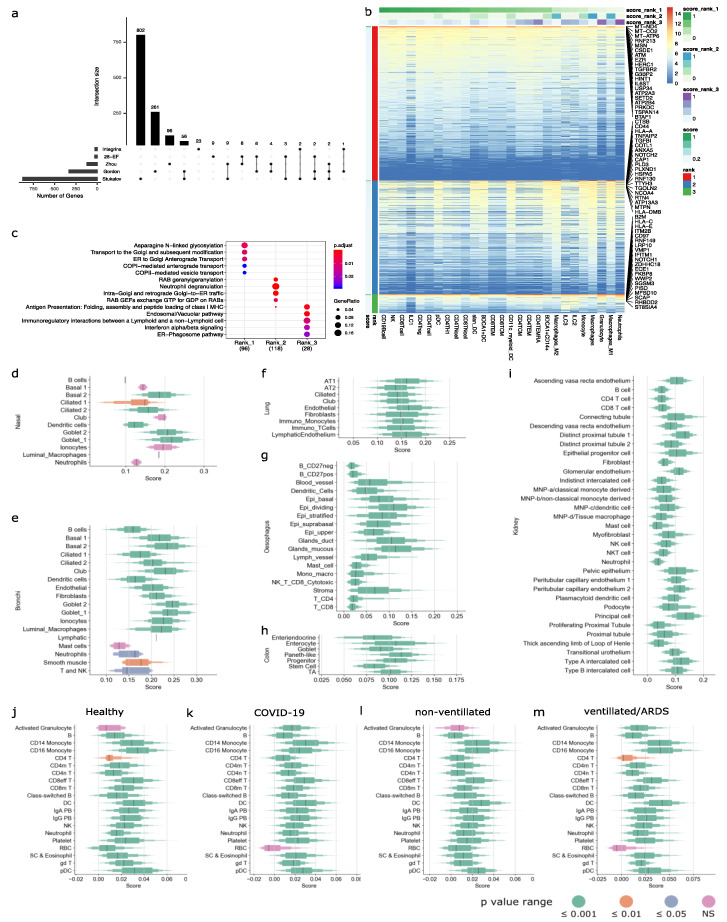
Enrichment of human lung cell line-derived virus–host interactome in circulating and tissue immune cells. (**a**) Intersection plot of different gene lists, shows size of the gene lists in bottom left panel, and overlapping size in the top panel. Overlapping genes between the gene lists are represented by vertical lines. (**b**) DIME heatmap showing ranked enrichment of Stukalov gene list in the circulating immune cells. The ranks depict the clusters as identified by DIME (see methods). Top 20 genes for each rank are shown. The cells are ordered based on the score of the rank 1 (top-weighted rank). Expression values are in log2(cpm + 1). (**c**) Pathway analysis (using Reactome) of the top 25 percent of genes of each rank, as identified by DIME. Boxplot showing the distribution of gene score of Stukalov genes for different cell types in the different tissue datasets of (**d**) nasal, (**e**) bronchi, (**f**) lung, (**g**) esophagus, (**h**) colon, and (**i**) kidney. Boxplot showing distribution of gene score of Stukalov genes in the PBMC dataset of (**j**) healthy controls, (**k**) all COVID-19 patients, (**l**) non-ventilated COVID-19 patients, and (**m**) COVID-19 patients that developed ARDS and were on ventilator. Black line represents median; height of box corresponds to number of cells in score range. Color of the box corresponds to the Wilcoxon *p* value computed with the alternative set to >0. See bottom right of Figure 2 for *p* value range.

Research on SARS-CoV-2 has been progressing at a rapid rate. During the course of our study, new data on the SARS-CoV-2 virus–host interactome emerged [36,50,51]. Among them, Stukalov et al. recently published the largest virus–host interactome profiles of SARS-CoV and SARS-CoV-2 using the A549 cell line (a human lung carcinoma cell line) [36]. We extracted specifically the SARS-CoV-2 interactome from Stukalov et al. and repeated the enrichment analysis as performed for the Gordon and Zhou gene lists (Figure 1, Figure 2, Figure 3 and Figure 4). The SARS-CoV-2-specific interactome from Stukalov et al. comprised 876 host proteins (Figure 5a). Despite having used similar methods (affinity purification and mass spectrometry) and having profiled the interactome of the same virus (SARS-CoV-2), the Stukalov and Gordon gene list shared only 60 genes between them (Figure 5a). However, Gordon et al. used the HEK293T cell line, while the Stukalov et al. study used the A549 cell line; perhaps the differences in the virus–host interactomes can be attributed to the difference in the cell line used by the two studies. Further research is warranted to explain the differences in the interactomes when different host cell lines are used. The 60 genes common between the Stukalov and Gordon gene list were associated with pathways such as glucosyltransferase activity (GO: 0046527), nucleoside–triphosphatase activity (GO: 0017111), purine nucleotide binding (GO: 0017076), etc. We then explored the circulating immune cell types that are enriched in the Stukalov gene list using the DIME analysis (Figure 5b,c). We found that several genes in the Stukalov gene list were enriched in lymphoid cells, such as B cells, NK, T cells, and ILC1 (Figure 5b, rank 1). These genes are associated with pathways such as asparagine N-linked glycosylation, transport to Golgi, COPI- and COPII-mediated transport (Figure 5c). N-linked glycosylation is a process taking place in the endoplasmic reticulum, where carbohydrate groups are attached (by glycosidic bonds) to proteins, while COPI and COPII are transport proteins involved in the transportation of proteins from the Golgi to the endoplasmic reticulum, where glycosylation occurs [52,53]. Glycosylation is crucial for “glycan shielding” of the S protein of the SARS-CoV-2, which enables the virus to evade the immune system by masking itself as self-glycans, a process that has been touted to be responsible for the enhanced infectivity of SARS-CoV-2 [54].

Furthermore, several other genes (Figure 5b, rank 2) in the Stukalov gene list were enriched in macrophages, and these genes are associated with pathways such as RAB geranylgeranylation, neutrophil degranulation, and Golgi transport (Figure 5c). Interestingly, the same pathways were also found in the Gordon gene list enrichment analysis (Figure S5b).

Next, we explored the enrichment of the Stukalov gene list in the single-cell RNA-Seq datasets of different tissues. Interestingly, tissue resident immune cells such as dendritic cells in nasal (Figure 5d), B cells, dendritic cells, and luminal macrophages in bronchi (Figure 5e), monocytes and T cells in lung (Figure 5f), several myeloid and lymphoid cells in the esophagus (Figure 5g), progenitor cells in colon (Figure 5h), and several lymphoid and myeloid cells in the kidney (Figure 5i) exhibited significantly positive gene scores for the Stukalov gene list. Thus, the data indicate that the SARS-CoV-2-interacting proteins identified by Stukalov et al. are expressed ubiquitously by tissue immune cells. The gene score plots for other tissue datasets can be found in Figure S11.

We then calculated the gene scores for the Stukalov gene list in the PBMCs of healthy controls and the different kinds of COVID-19-positive patients (Figure 5j–m). We observed that, as shown for the Gordon and Zhou gene lists (Figure 4e–h), myeloid cells such as dendritic cells, CD14 and CD16 monocytes, and plasmacytoid dendritic cells, and lymphoid cells such as CD8 effector T cells and gamma delta T cells, had high gene scores across all types of patients. We observed that for activated granulocytes, the gene scores were significantly positive only in patients who developed ARDS and were on ventilator (Figure 5m).

## 4. Discussion

Understanding the pathophysiology and target cell populations is crucial for understanding the mechanism of action of SARS-CoV-2 and developing therapies for COVID-19. Here we used publicly available single-cell RNA-Seq datasets from the COVID-19 cell atlas [23,48] to study which cells express ACE2 receptor and whether CoV (SARS-CoV-2 and other CoVs)-associated genes are enriched in those cell types. We found that a tiny fraction of cells in tissues that are known to be targeted by SARS-CoV-2 express the ACE2 gene, especially the lungs. In corroboration with Hou et al., we found a decreasing gradient in the fraction of ACE-expressing cells from proximal to distal respiratory tract [15]. A small fraction of ACE2-expressing immune cells were found in several tissues. Additionally, these immune cells in circulation and in the tissues were enriched in the SARS-CoV-2-associated genes.

Interestingly, circulating immune cells do not express the ACE2 gene as shown in bulk transcriptome analysis. Multiple reasons can explain this finding. First, we used bulk transcriptome data for circulating cells and hence we lost single-cell resolution. However, we used bulk transcriptome data to study multiple conventional circulating cell types. Second, it could be that ACE2 expression is induced when the immune cells reside or reach different tissues and that ACE2 is not expressed when the immune cells are circulating. This is supported by the fact that multiple other cell types (such as enterocytes, endothelial cells, fibroblasts, etc.) show differences in terms of ACE2 expression in different tissues. Last, the level of ACE2 expression in immune cells could be low and/or could differ between cell and tissue types. We did not find this to be the case (Figure S6), as ACE2-positive immune cells typically expressed the ACE2 gene at a level that was comparable with the other SARS-CoV-2 target cells found in the corresponding tissues.

Additionally, we used a publicly available single-cell RNA-Seq dataset of PBMCs from COVID-19-positive patients to explore the expression of SARS-CoV-2-associated host factors (Figure 4 and Figure S9). We found that only a tiny fraction of cells expressed ACE2 and TMPRSS2. Interestingly, the fraction of ACE2- and TMPRSS2-expressing cells was found to be heterogenous across the different patients and was not representative of severity of disease or clinical outcome. However, on inspection of the SARS-CoV-2-associated host factors, we found that several immune cells (such as dendritic cells, plasmacytoid dendritic cells, monocytes, T cells, etc.) were enriched similarly across the different COVID-19 patients, indicating that ACE2 and TMPRSS2 may be insufficient to study SARS-CoV-2 infection.

Alternatively, some studies have indicated that SARS-CoV-2 might use additional receptors and co-receptors for infection [33,35,55]. Consequently, we reperformed the entire analysis using entry factors compiled by Singh et al. (28-EF gene list) and on all known integrins [34]. We found that most of the immune cells in all tissues are enriched in the 28-EF gene list, while the integrin gene list was enriched in only certain tissues, such as alveoli, bronchi, lung, colon, esophagus, pancreas, and testis (Figure S3). We found that all circulating immune cells express RHOA (Figure S4a), a member of the Rho-GTPase complex that interacts with SARS-CoV-2 structural proteins [27]. Circulating myeloid cells such as granulocytes, dendritic cells, and macrophages were enriched for most of the 28-EF gene list according to DIME (Figure S4a). Among the integrins, we found integrins such as ITGA4, ITGA5, ITGA6, ITGAL, ITGAM, ITGAX, ITGB1, and ITGB2 expressed by all circulating immune cells; lymphoid cells such as Th1, NK, and TEMRA and myeloid cells such as dendritic cells, granulocytes, and macrophages were enriched for the integrin gene list according to DIME (Figure S4b). In the PBMCs of the COVID-19-positive patients, immune cells such as monocytes, dendritic cells, and neutrophils are enriched in the 28-EF gene list; monocytes and platelets are enriched in the integrin gene list (Figure S10).

In this manuscript, we have limited analyses to gene lists comprising entry factors and intracellular interacting proteins from five published studies; however, our approach could be extended to exploring genes that regulate these entry factors, for example, by exploring the EMILIN family proteins that may regulate FURIN activity [22]. As more studies become available on the regulation of multiple such entry factors, our approach and tools [39] can be extended to further explore these genes.

Given our computational findings, we propose that further studies should be performed to test if tissue immune cells can express ACE2 and TMPRSS2 genes. Studying primary immune cells in circulation may not be sufficient to study ACE2 expression in immune cells. Recent published and preprint studies have shown that a few immune cell types (such as T cells, B cells, monocytes, and macrophages) in tissues such as the lung, lymph nodes, and spleen are susceptible to SARS-CoV-2 infection [30,31,32]. However, the immune cell types (including ACE2 cells) susceptible to infection in all the other tissues remain uncharted. If even a small fraction of activated immune cells such as T cells and dendritic cells can get directly infected by SARS-CoV-2, they might help the virus to migrate from one tissue to another. Thus, we propose studying host factors and immune cells that may play a role in viral replication, assembly, and infection. Further research is needed to explore if SARS-CoV-2 can directly infect circulating and tissue immune cells.

## Data Availability

All data analyzed in this study has been referenced in the methods.

## Supplementary Materials

The following are available online at https://www.mdpi.com/article/10.3390/v13091757/s1, Figure S1: Fraction plots of ACE2 and TMPRSS2. Figure S2: Intersection plot of different gene lists. Figure S3: Gene score plots for different tissues using gene lists from Zhou, Gordon, 28-EF and Integrins. Figure S4: DIME heatmap showing ranked enrichment of 28-EF and integrin gene lists. Figure S5: Pathway analysis of top genes as identified by DIME for Zhou, Gordon, 28-EF and Integrin gene lists. Figure S6: ACE2 receptor gene expression of different cells across bronchi and lung. Figure S7: Heatmap of protein expression of circulating immune cells from the immprot database. Figure S8: Heatmap of protein expression of different cells from 49 tissues taken from the Human Protein Atlas (HPA). Figure S9: Enrichment of SARS-CoV-2 host factors in PBMCs of COVID-19 patients from Wilk et al. dataset. Figure S10: Enrichment of SARS-CoV-2 host factors in PBMCs of healthy and different types of COVID-19 patients taken from Wilk et al. dataset. Figure S11: Gene score plots for different tissues using Stukalov gene list.
